# Emotional cycles and collective action: Global crises and the World Social Forum

**DOI:** 10.1057/s41282-022-00302-3

**Published:** 2022-09-19

**Authors:** Giuseppe Caruso

**Affiliations:** Faculty of Social Sciences, University of Helsinki, Helsinki, Finland

**Keywords:** global justice movement, emotional sequences, World Social Forum, global financial crisis, Wilfred Bion, collective action

## Abstract

As the world suffers the effects of the Covid-19 pandemic, global justice activists pursue political solutions to its devastating consequences especially on the weakest sections of the world’s population. I analyse activists’ responses to the 2008 financial crisis to reflect on how collective action is impacted by social crises. The global justice movement and the financial sector face recurring, intertwined, and inversely related cycles of exuberance and crash. I find that, on the one hand, the prevalence and intensity of recurring crises in large transnational collective actors depend on factors including their prevalent emotional dynamics, their dispositions towards their objectives, and their ability to gauge external reality. On the other hand, differential outcomes of crises in groups are accounted for by the capacity to mourn the losses suffered, as opposed to the denial of responsibility and the externalisation of blame. I analyse these emotional dynamics through psychoanalytic lenses to provide a contribution to the literature on the cycles of collective action and, more broadly, to the study of political action and social change.

## Introduction

As the world suffers the effects of the Covid-19 pandemic, global justice activists pursue political solutions to its devastating consequences. In this paper, I study activists’ responses to the 2008 financial crisis to draw insights on whether political collective action can help manage and work through the effects of global crises. While its immediate consequences were felt in Wall Street and the City of London, they soon became global. The evictions, unemployment, hunger, bankruptcies, suicides that followed affected disproportionately the most vulnerable sections of the world’s population. Consider, for instance, the analysis published by the World Bank in October 2009: “When you have these kinds of growth slowdowns, infants die. … For the size of this slowdown in Africa, you could lose about 30,000 to 50,000 infants before their first birthday” (World Bank, 2009). This resonates with tragic currency as the world economy slowed down due to the Covid-19 pandemic (World Bank, 2020).

When the financial crisis struck, the World Social Forum (WSF), arguably the largest global civil society initiative to date, mobilised to advocate for profound changes to the social and institutional structures that caused it. WSF activists faced comparable crises in previous circumstances. The forum’s emergence in 2001 followed the 1999 demonstrations in Seattle against the World Trade Organization (WTO), and against the International Monetary Fund (IMF), the World Bank, and the G8 in 2000–2001. That wave of global protests was a reaction to the social impact of the Asian financial crisis in 1997 and of the dot.com crash in 2000. WSF activists attributed the social and human consequences of global financial crises to the global prevalence of neoliberalism, an ideology, they claimed, built on domination, exploitation, and a culture of denial and blame. WSF’s opposition to neoliberalism was enshrined in its Charter of Principles that it adopted in 2001. To “neoliberalism and the domination of the world by capital and any form of imperialism”, the WSF opposed a commitment to “building a global society directed towards fruitful relationships among people and between humankind and the planet” (WSFTE, n.d.). The stress on the importance of a caring relationship with the environment has become increasingly current in the face of the prevailing global denial of the consequences of climate change, including its effects on public health and the spread of viral pandemics (see Weintrobe, 2021; Hoggett, 2020).

Here, I limit my analysis to WSF’s International Council (IC). I have worked with the WSF since 2002, and have been a member of its IC since 2009, though since 2015 I have been less active. The present paper focuses on the IC meetings that took place in Rabat (May 2009), Montreal (October 2009), Mexico City (2010), Dakar (2011), and Tunis (2013). The IC has a mandate to facilitate the WSF process by helping raise resources and supporting the organisers of the global events. It also decides the location of these events. Its membership included over 200 transnational organisations and networks, though not all have been involved to the same extent. Lately, the active membership is limited to a few dozen trade unions, peasant movements, and human rights and development NGOs. Hundreds of activists participated in the meetings considered, from virtually the whole planet, though the majority were Latin American and European activists.

The WSF does not claim to represent the entire global justice movement. Yet it is arguably a paradigmatic example of it (Teivainen, 2012, 2016; Caruso, 2012, 2017a, 2017b; Conway, 2012). Since 2001, its global events have attracted hundreds of thousands of participants from all over the world. It has since inspired dozens of local, national, regional and thematic social forums that have attracted, over two decades, millions of participants. I recognise that the global justice movement is organised in structures of great complexity that make their treatment in the singular or their representation by one of its components, however prominent and sizeable, untenable. A conflation of different voices into a singular collective one would lead to forms of epistemic obliteration. However, emotional dynamics like those considered here show how behavioural convergences develop in very large groups like the global justice movement. This makes it possible to consider such groups as coherent, if enormously complex, wholes (Gould, 2009; Burack, 2004; Alford, 1994; Bion, 1961) in which emotional variations exhibit some predictability.

Social movements develop through ebbs and flows of mobilisation that are influenced by both internal and external, social and political, factors and by material, symbolic and emotional dynamics. I focus on WSF’s emotional dynamics and their effects on its ability to pursue its objectives. My main contention is that the effectiveness of political collective action is diminished by the pressure of unacknowledged or unmanaged emotions. Uncontained emotions affect the group’s membership, prevent collaboration between its members, and lead to its break-up. The structure of my argument is two-pronged. First, I show how patterns of organisational ebbs and flows in large social movements like the WSF unfold as recognisable emotional cycles. Then, I argue that the study of these cycles through a psychoanalytic lens can contribute unique insights to the literature on the cycles of collective action and their structural and political (McAdam et al., 2001; Tilly et al., 2020; Tarrow, 2011), cultural and narrative (Polletta, 2006; Staggenborg, 1998), frames-related (Snow & Benford, 1992), and social psychological components (van Stekelenburg & Klandermans, 2013; Melucci, 1996). The broader implication of these findings is that studying cycles of collective action through psychoanalytic lenses can shed light on wider political dynamics and social change. My field of reference is the emotional turn in the social sciences, which I briefly introduce in the remainder of this introduction. The rest of the paper is structured as follows: in section 2, I describe the emotional dynamics in the WSF IC following the financial crisis; in section 3, I analyse WSF’s emotional cycles; section 4 concludes.

After the postwar prevalence of research on individual and group actors’ rationality, from the mid-1990s there has been a “return of the repressed” in political and social studies (Goodwin et al., 2000). Emotions in collective action have since received growing attention (Van Ness & Summers-Effler, 2019; Hoggert & Thompson, 2012; Jasper, 2011; Flam & King, 2005; Goodwin et al., 2001). Social movement actors are not seen as merely rational calculators, nor are emotions considered expressions of a primordial component of our human nature superseded by rational cognition. Instead, emotions are understood as key motivators of group dynamics and sophisticated advisors in both individual and collective decision-making. They form the bases of political alliances, identities and affinity construction (Gould, 2009; Juris, 2014). Feminist and queer scholars and activists provided a crucial contribution to the development of the new mood about emotions. They criticised the hegemonic social theory for marginalising the role of emotions in social and political life and their alleged privileged carriers, women and queer people (as opposed to rational men). They deconstructed activists’ behaviour and showed the centrality of emotions in decision-making (Taylor & Rupp, 2002; Hercus, 1999; Whittier, 2021). This emotional turn has often been inspired by cognitive psychology. This led to a limiting cognitive bias (Thompson & Hoggert, 2012; Gould, 2009, 2012; Turner, 2006; Barbalet, 2002). Deborah Gould in her powerful and moving account of ACT UP, noted that

scholars in the emotional turn sometimes have overly cognitivized and rationalized political feelings and downplayed what I call affect. … In my view, if we neglect affect and fold feelings into cognition, or emphasize the cognitive dimension of feelings and how individuals’ feelings align with their reason, we not only lose sight of the bodily, visceral qualities of feelings, but we also obscure a number of insights that an affective ontology provides for understanding political action and inaction. (2009, p. 23)

Gould’s work is a compelling example of psychoanalytically informed social research.^1^ For her, emotional cycles inform activist mobilisation and demobilisation. Similarly, from the point of view of the theory used here, “overly cognitivized” approaches downplay the causal emotional processes leading to collective decision-making and action. Consider also Jasper’s analysis: while warning to beware the Scylla and Charybdis “of emotions as automatic bodily disturbances or as an overly calculating reflexive awareness,” he recalls how “for decades, psychoanalysis had offered the only serious toolkit for talking about emotions in politics” but “its promise faded in the 1970s and 1980s, as cognitive psychology developed as an alternative” (2011, p. 288). I claim, instead, that a psychoanalytic understanding of emotions is crucial to understand individual and group motivations and behaviour. Psychoanalysis provides unique insight into emotional cycles, whereas the behavioural expression of emotions may be insufficient to explain their prevalence, articulation, and transformations. My contention is that, by paying attention to emotional cycles and their unconscious dynamics, it is possible to better theorise the development of collective political action and behavioural enactments. A psychoanalytically informed social science can, therefore, theorise the interplay between social structures, group dynamics, and individual motivation (Turner, 2011; Gould, 2009; Smelser, 1998).

## Activists in (the) Crisis

In this section, I recount the difficulties encountered by the WSF IC activists as they tried to work through the mixed feelings caused by the financial crisis and by the global justice movement responses to it. Feelings of loss and anger, but also of opportunity and responsibility, generated powerful emotional group dynamics. Before I start, I need to flag two important matters. The first is, that given the space available here, the illustrations I give below can only present a sketch of the dynamics I discuss, but hopefully a vivid enough one. The second, more substantive matter, is that the emotional cycles I discuss do not manifest themselves in separate movements but are tightly intertwined. Undercurrents of anger and elation, guilt and promise, despair and hope are present, and often express themselves at the same time. In what follows, I highlight prevalent moods and their development over time.

The first global WSF after the 2008 financial crisis took place in Belém, Brazil in January 2009. The mood of the over 100,000 participants, saddened and angered by the consequences of the crisis on the most vulnerable sections of the world’s population, was otherwise upbeat, triumphant on occasions. According to many of those participants, the crisis was an opportunity to drive democratic transformations and even overcome capitalism. These were widely shared feelings in the global justice movement. Consider Paul Mason’s words: “Basically, neoliberalism is over: as an ideology, as an economic model. … Those who want to impose social justice and sustainability have a once-in-a-century chance” (2009, p. vii). The radical Midnight Notes Collective wrote, along similar lines, “for Marx and his comrades the approach of a crisis was closely watched with much excitement, even glee, since it signalled to them the possibility of a revolution. … It is with this knowledge, from this perspective, and with a cautious joy that we approach the present crisis” (2009, p. 3). These words resonate with those of various IC members, such as: “Belém has been the most important edition after 2001. Because of the crisis, we are able to suggest ways out of the crisis” (1, Rabat).^2^ Like the exhilaration accompanying the first WSF in 2001, this enthusiasm reinforced the belief that WSF's vision could soon be fulfilled.

Anticipation of future achievements is an important element of human motivation, especially in the field of global justice activism. With the perceived opportunity, came also a feeling of heightened responsibility. “[If] Belém was a wake-up call to deliver that alternative, from here we have to show that we are able to deliver that alternative” (2, Rabat). On the other hand, “we have a responsibility; the movements are demanding that the WSF offers a way forward” (3, Rabat). As the IC debated WSF's strategy, world leaders spoke with confidence at the G20 meetings in London and Pittsburgh (in April and September 2009). They declared the crisis over and implemented Keynesian policies to support the global recovery. It seemed as though the state of dismay following the crash had been superseded and that a solution was in sight. The IC, at its 2009 meetings in May in Rabat and in October in Montreal, reacted with a mixture of astonishment and self-criticism soon turning into a generalised sense of powerlessness and defeat. The global crisis risked to become, according to many activists, a terminal crisis of the WSF because it had failed to take advantage of this unique opportunity to foster change. In Montreal, frustration and a sense of defeat began to prevail. Consider the following: “I think we are missing the opportunity to respond to the crisis but I think that there is still the possibility to build an alternative. Today the debate is about if the crisis is over or not. So we need to discuss how, even if the crisis is over, the world is anyway based on a crisis, on critical foundations” (4, Montreal). The outcome of such tensions between global capitalism and the movements was that “there is so much more pessimism than in Rabat. Because capitalism has shown its resilience and the Left has not been successful in taking advantage of the crisis” (5, Montreal).

The perceived opportunity spurred a sense of urgency, which, in turn, generated growing anxiety that the window of opportunity was closing. Anxiety turned into frustration as IC activists felt they could not achieve their goals. Somebody burst out that “we are not able to better organise this debate on the crisis!” (6, Rabat). At a later meeting in Mexico City in May 2010, somebody else said, “We pretend to be an alternative pole of convergence but we really have no idea what that convergence would be. While we are very good at talking, we have no idea how to design a strategy and implement it” (7, Mexico). This frustration turned onto itself as activists felt responsible for their failure to act effectively. Some felt the burden of guilt towards those that they represented: “When I go back to my people I want to know what to tell them when they ask how we managed to strengthen their struggles” (8, Montreal). With guilt, a sense of losing touch with reality intervened; “the IC is removed from the reality of the drama of the people affected by the crisis” (9, Montreal).

Anxiety, frustration, and guilt strengthened by the apparent success of their adversaries, generated a deep feeling of failure among activists. The growing sense of defeat turned into a deep sense of irrelevance, “the WSF is at a crossroad between relevance and irrelevance” (10, Rabat). “There was a time,” an IC member reminisced, “when the Forum was relevant, when people would come together to talk about their common strategy, this is not anymore. We don’t have a common vision, indeed we do not have any vision at all. If we do not have vision we are destined to irrelevance” (11, Montreal). The global crisis, therefore, “is not only a manifestation of the problems of capitalism but it is also a crisis of the Left because it has not challenged capitalism coherently and strongly enough to the present day” (12, Montreal). Such was the sense of irrelevance that “if the crisis is systemic then the WSF has no meaning because revolts against that system will follow internal logics on which the Forum has no influence” (13, Rabat).

Activists recognised their crisis and they tried to make sense not only of its external causes, but also its internal nature. In the process, they became aware of the entangled relationship between activists and their adversaries, between internal and external causes, between imbalances of power and their embodiment in the global subalterns. Someone suggested that progressive movements were undermined by the economic crisis as “our funds depend from the wealth of funders,” which, in turn, “increased the competition between organisations, reducing trust and increasing suspicion” (14, Rabat). However, the fragmentation of the global justice movement was not only due to lack of resources. It was a “major crisis of working together” (15, Montreal) that would also affect future mobilisations as the WSF was “going through a deep crisis of credibility” (16, Montreal). While the movement articulation into separate sectors had been its main strength (under the slogan “unity in diversity”), the dispersion of global mobilisations and the reduction in funding opportunities increased the competition among those sectors (i.e. environmentalists, feminists, labour, human rights, etc.). Indeed, the many events of the global WSF process were “in competition one against the other as we cannot go to all the forums” (17, Rabat).

To illustrate the predicaments faced by the IC, the WSF and the global movement, an activist presented a striking image of failure and despair. “The Paris uprising [of 2005] is paradigmatic. Resistance had no slogan as if it had lost the words to express itself. Even at the beginning of the Second Intifada, this group inside Israel went to the streets with no slogans. We are losing our capacity to express our political resistance, and maybe this is the reason of fragmentation, and maybe this has to do with depoliticisation. We cannot only blame the system as the cause of our fragmentation. Maybe we need to criticise our way of organising” (18, Montreal). This is a powerful image. Slogan-less, almost speechless individuals, stunned by injustice yet unable to put words to their demands. Unable to symbolise the reality they were affected by, they could only drag themselves in the streets either in silence or making inarticulate sounds, ineffective chants. Or riot, as this speechless helplessness, this impotence, could find expression only through the pained and painful destruction of things. Acknowledging their speechlessness, activists reintrojected blame. Perhaps, they said, we should not blame the system, our adversaries. Instead, we should recognise our own difficulties.

Blame remained central, but changed direction. Not only pointing exclusively at global finance and neo-imperialism, it searched for the the cause of activists’ fragmentation and speechlessness. The erosion of trust undermined the possibility to speak to each other, to find a voice as a movement, to “speak” to power. This speechlessness, this powerlessness, prevented activists from working together, which in turn made them feel even more isolated and helpless. Someone said, “We do not trust each other, people do not trust anyone in the Forum and that’s why others are not here anymore. There is no trust, no vision, no ideology” (19, Montreal). Later, he added, “We have lost the confidence to fight; this is because we are not clear about what is an alternative to capitalism and this is the crisis of the resistance” (20, Montreal). A participant suggested that the frustration in the IC was due to confusion between “the four levels of the WSF: WSF as space where encounter happens; as framework where there is an agenda setting exercise …; as process, the expansion of the forum itself; and as actor that takes action” (21, Rabat). This confusion generated exhausting procedural negotiations within the IC and together caused scepticism and withdrawal among members.

Whereas procedural issues are at the core of the WSF's culture of politics, its workings often become confused and frustrating for members in moments of crisis. Sometimes, the contrast between being a symposium of ideas in the face of the actual consequences of global crises was too stark not to affect activists’ emotions and self-perception. In these circumstances, blame was first taken in by the group and then externalised again onto its institutional arrangements and political identity. The perception by a section of the WSF of its inability to express itself politically increased the emotional tone of internal arguments. Feelings of impotence, in turn, generated self-representations of irrelevance, mistrust and withdrawal. In this climate, activists called for a reconsideration of WSF’s role in global transformation: “it is not true that we do not offer alternatives, but the WSF is not the right actor to take ahead those initiatives” (22, Montreal). This feeling of despair was caused by the realisation that financial capitalism had not capitulated; indeed, it had resumed business as usual. The crisis of financial capitalism became the crisis of the WSF, which, in turn, was representative of the general crisis of the global justice moment. This crisis expressed itself through fragmentation, internal competition and distrust, lack of democracy and vision, strategic incoherence. Frustration was so high that at the end of the Rabat meeting someone exclaimed, cutting through raised voices, impatience and irritation, “But why, the ending of all our meetings are so heated” (23, Rabat).

Solutions were discussed, attempts were made to revive smouldering debates, to stoke hopes and encourage disheartened fellow activists. Activists made an attempt to recover the earlier upbeat feelings by creating a mobilising momentum towards the Copenhagen conference on climate change in December 2009. They proposed to protest against the creation of a global carbon market. Instead, someone proposed that “we should talk about a real carbon market that avoids a subprime crash in that market as well” (24, Rabat). More generally, and more importantly, it was felt that the Forum should return to focus on creating opportunities for the fragmented global justice movement to connect and work together. The WSF driving vision, after all, is that it “should provide the opportunity to the Left to present on the global stage a larger coherence in terms of the alternatives it suggests. I know that the open space has been a celebration of differences but I think that now the time has come that we also talk about what we have in common” (25, Montreal). If that could be achieved, “if we put our foot in the window … we might keep it open” (26, Montreal).

New ideas to reinvigorate the WSF process were discussed in Mexico City. The slogan of the Mexican Social Forum, taking place alongside the IC meeting, was “Other ways out of the crisis are possible”. At that meeting, South American movements proposed a *buen vivir* (good living) agenda. African activists, instead, proposed “a new universality” as the driving principle of WSF 2011 to take place in Senegal. These visions addressed the crisis pointing out the shortcomings of global development. However, they did not anticipate change to derive from the regulation of its internal dynamics. Rather, radically at odds with the global development discourse, they suggested looking to postcolonial, post-patriarchal, and antiracist sources of development in harmony with the environment. *Buen vivir* was “an entirely different paradigm vis-a-vis growth and de-growth” challenging “the crisis of civilisation of the capitalist modernity” (27, Rabat) and aimed “to make a kind of cultural revolution” (28, Rabat and Montreal). The vision for a “new universality” stressed that, as Western modernity was built on colonialism, slavery, capitalism, imperialism and the hopeful but often enslaving rationality of the Enlightenment, the WSF would adopt a cosmopolitan and emancipatory vision based on the values of hospitality, conviviality and solidarity to counter the uncompromising individualism and competition at the heart of capitalism. The new universality was eventually overshadowed in the 2011 Dakar WSF by the North African revolts on which WSF's hopes converged at once. A new euphoria took over the global justice movement as the so-called Arab Spring swept through North Africa and inspired the occupation of squares in every region of the planet.

In the following years, the shortcomings of those rebellions, widely discussed in the 2013 Tunis WSF, undermined, again, the activists’ confidence and elicited new hopelessness. It was in Tunis, that Chico Whitaker, one of WSF’s founders, dramatically suggested that the IC crisis might be beyond redemption. He claimed that the IC was a white elephant in need of euthanasia. Disaffection and frustration were dampening activists' participation in the IC, and there was a fear of this contagion spreading to the WSF as a whole. The contagion did indeed spread to the WSF as it struggled to sustain its relevance in the following years. From the perspective of this article, Whitaker’s words illustrate the potentially deadening effects on global activism of failing to work through its own crises. It is an undeniable fact that power imbalances are overwhelming against global justice activists and their subaltern voices are actively silenced. However, it is also important to observe internal group dynamics when reflecting on the activists’ self-analyses of ineffectiveness. Whereas overwhelming power does frustrate attempts at emancipation, internal group dynamics too create frustration, hopelessness and demobilisation. As shown above, activists moved from one object of emotional investment to the next, transferring onto the new one their hopes and expectations. These rapid and intense investments, however understandable and part of social movement mobilisation, raise questions about the working through of previous crises, the realistic approach to the present, and the possibility of the repetition of known emotional sequences from infatuation to crash. Fast shifts from one *phantastic* object to the next (see below) and from crisis to euphoria, ignite recurrent crises in the global justice movement inflated, perhaps, by the accumulation of unattended conflicts. This experience closely recalls current investments in the WSF as an alternative catalyser of political action following the Covid-19 pandemic. “Another World is Possible”, WSF’s slogan, rings suggestive and urgent to global activists, but the possibility of disappointment and despair looms large as investments in social justice and change are sometimes not grounded in reality and difficult to sustain in the face of inevitable setbacks and powerful resistance.

## (Psycho)analysis of Emotional Sequences

A succession of intense emotional waves affected the WSF and indeed the global justice movement following the global financial crisis. When the crisis began to affect the most vulnerable sections of the world’s population, activists felt angered and distraught. Later, as they convened in large numbers, unity was felt like strength, it generated hope and exhilaration. Recall the excitement of the participants of the Belém forum mentioned above. The delay in achieving the ambitious objectives that activists set for themselves and the vigorous measures implemented by national governments and global financial institutions, caused disappointment among activists and disabling feelings of powerlessness and irrelevance. The consequences of the fantasy of a dying capitalism were soon felt. Reality cannot forever be denied and eventually makes a sudden and unexpected return. Unexpected, because its elision had become so complete that its true forms had become both forgotten and inconceivable. The repressed returned, and frustration and shame engulfed those who lost themselves in the collective euphoria. When the repressed aspects of reality return, the split anxiety turns to panic and the object, onto which the group had placed, as it were, all its emotional eggs, becomes persecutory and elicits murderous drives like those expressed by the euthanasia comment. These feelings led to increasing disengagement. Pushed by its own growing weight, this disengagement spiralled downwards leading to a fragmentation of the global justice movement as the activists turned their political action from the global scene (the WSF) to the national and local context (the so-called square movements).

The study of emotional dynamics, like those described above, offers powerful tools to complement theories of historical sequences in collective action (Kriesi et al., 2019; McAdam & Tarrow, 2019; Tarrow, 2011; McAdam et al., 2001). More ambitiously still, the study of emotional dynamics in very large groups can complement structural and political analyses of society and change by adding an increased sensibility to both individual and group unconscious motivations and conflicts. Seen from this perspective, the global justice movement is an extended group whose members broadly identify with a shared vision of social justice and equality against the global economic and institutional hegemony of capitalism. They share a history and, in some sense, membership in a global underclass. In the case of the WSF, powerful emotional dynamics developed around its slogan “Another World is Possible”, its open-space methodology, its more or less identifiable group of founders and leaders, and the values and political intents enshrined in its Charter of Principles. Those emotional dynamics caused the group to become entangled in internal dynamics that subtracted energy from the fulfilment of its goals. How can we make sense of the dynamics underlying these emotional cycles? To begin addressing these questions, I turn to Wilfred Bion’s theory of group dynamics and, later, to that of David Tuckett, who built on and developed Bion’s work.

The complexity of Bion’s (1961, 1970) work can hardly be done justice to here. Yet, some of its core concepts can be summarised to be used in what follows. Bion described two kinds of group dynamics, which he called the work group (W) and the basic assumption group (BA). The former is attuned to reality, it has conscious purposes and elicits collaborative action to meet its objectives. The latter is dominated by wishful thinking, is turned inwards on existential, bureaucratic and procedural matters, which absorb most of its energy and cause conflict and fragmentation. To be sure, W and BA indicate emotional configurations simultaneously active in groups. The prevalence of either defines the overall state of the group, its relationship with reality, its objective, and the relationships between its members. Groups cycle between prevailing W and BA modes. Work dispositions recede as basic assumptions advance and wishful thinking overwhelms the group’s reality-based judgement. Wishful thinking motivates unrealistic decision-making processes in which uncertainty is denied (like when risky speculative aspects of financial investments are split off and securitised through derivative vehicles that provide the illusion of conquering uncertainty). The knowledge that could elicit a more balanced decision-making is denied and with it the anxiety caused by uncertainty. Realistic ambivalence towards the group’s objectives and the conflicts (internal to the individual and between group members) that both ambivalence and anxiety introduce into decision-making are excised and unconsciously expelled. When this process is completed, and only a one-sided perspective remains accessible to consciousness, action can be pursued without the impediments of doubt and an attitude of complete abandonment.

Building on these premises, David Tuckett studied financial crises to understand how institutional settings, group dynamics and mental states generate path-dependent emotional sequences in the global financial sector. The emotional sequences he analysed develop from “excitement to mania to manic defence (unease) to panic to shame to blame or mourning” (Tuckett, 2011, p. 16). The last stage of the sequence is crucial to learning from experience and differentiates blame-laden attitudes of denial from mourning for the loss suffered (including of one’s self-worth as a consequence of failure). Without mourning the death of the “white elephant”, as analysed in the previous section, organisational development is impossible. If the loss cannot be tolerated, triumphant mechanisms (manic defences) prevail. The euthanasia, in that case, could be interpreted as spiteful retaliation for the disappointment the group (in this case, the WSF IC) had caused to its members. Failing to work through the emotions associated with loss, condemns the WSF and the wider global justice movement to cycles of compulsive repetition (see Freud, 1914/1958, and Klein, 1940). When excited investments into powerfully desired objects concentrate across large (even transnational) groups, they become vulnerable because of this very convergence (imagine the effect of the passengers on a boat moving all at once to one side to admire an arresting sunset). Even in the face of recognised vulnerability, systems often fail to correct their trajectory. This failure to see first and act later when recognition strikes, generates a self-fulfilling belief in apparently unstoppable cycles.

To simplify, a work group state, in which conflicting aspects of an emotional investment are kept together and drive the conflicted engagement/disengagement decision-making process, helps to contain the anxiety generated by the radical impossibility of predicting the outcome of the decision taken. A prevailing basic assumption group separates away risk, doubt, anxiety and uncertainty from awareness. When this happens, both individual and group decisions assume a sense of necessity, of inevitability. Fast decision-making feeds its own confidence and it becomes euphoric as thinking and deliberation are bypassed. Tuckett describes this abandonment and loss of the sense of reality as similar to feelings and behaviour in the initial phases of falling in love, when individuals are sometimes said to be “madly” in love. Objects of emotional investment, related to in this manner, assume the character of “phantastic objects”, their existence legitimated by what Tuckett calls a self-reinforcing “conviction narrative” (see below). Phantastic objects in Tuckett’s formulation are “subjectively very attractive ‘objects’ (people, ideas, or things) which we find highly exciting and idealise, imagining (feeling rather than thinking) they can satisfy our deepest desires, the meaning of which we are only partially aware” (2011, p. xi).

In the unrestrained embracing of a phantastic object, a crucial role is played by what Chong and Tuckett (2015) call “conviction narratives”, deployed to make sense of the choices actors make. Conviction narratives are complex stories mobilised by individual and collective actors in order to explain and justify their decisions. They are a combination of elements that either attract towards an object or help repel doubts about commitment. These narratives allow them to manage radical uncertainty about the future outcome of their decisions (Chong & Tuckett, 2015; Johnson et al., 2022). These narratives, among other things, consolidate the belief that something exceptional is happening that gives the world a radically different outlook, a different internal logic and different dynamics. Narratives of exceptionality are highly contagious in social contexts in which the basic assumption group mentality prevails and, in turn, they contribute to its consolidation. Eventually a critical mass believes in the narrative and joins in causing large behavioural waves. When applied to the global justice movement, this is how this theory looks like.

Social movements, NGOs, community organisations, academics and journalists became quickly captivated by the idea that the WSF “open space” had finally allowed the global justice movement to work through its weaknesses, fragmentation, internal power dynamics, and the mistrust between its members. For the activists, the Battle of Seattle against the WTO had established, the Prague demonstrations against the World Bank and the IMF had proved, and the protests in Genoa against the G8 had confirmed that unity (in diversity) constituted not only a righteous but also an invincible strength. The WSF, as a prefiguration of the idea of another possible world, became immediately the obvious corollary to this narrative. Change was not only possible, it was already here and now. The (good) excitement remained conscious while the (spoiling) knowledge that things were not so simple was soon overwhelmed by statements like that in the *New York Times* declaring the global justice movement the second world superpower (Tyler, 2003). A similar emotional investment took place after the global financial crisis in the occasion of the Belém WSF as I described above.

Objects, in themselves neither good nor bad (or, more precisely, both good and bad at the same time), take on phantastic qualities because of the expectations that actors have in them, of the relationships they build with those objects, and of the dynamics developing among them when in relation with those objects. Those expectations and relations can develop attributes of unreality and destructiveness. Credit swaps were useful financial tools, but the collective unrealistic belief that they eliminated uncertainty from financial markets transformed them into objects of powerful destructive group dynamics. Similarly, the WSF had not inherently phantastic qualities (it has been indeed successful at mobilising millions of global activists). What transformed it into a phantastic object was the belief that it could be (here and now), if activists related to it with abandonment and loyalty, the other world they wished. This is the crucial passage. Loyalty replaced the thinking necessary for the reality checks required to appreciate both the commitment and the work needed to transform potentiality into actuality, and the external obstacles preventing the realisation of the group goals including, in this case, overwhelming global power imbalances and the objective difficulty of surpassing capitalism.

Understanding the relationships that a group establishes with its objects can help assess whether its goals and activities are realistic or whether a state of euphoric or depressed unthinking prevails. In the present context, it can help assess whether a collective demonstration of indignation, like the activist responses to the human and social consequences of the financial crisis, can achieve its goals or whether they are built on unrealistic beliefs and expectations. During the euphoric excitement following the 2009 WSF, reality seemed to move to the background. The activists’ understanding of what was possible, desirable, and likely to happen changed substantially due to a shift in collective mental attitudes. In those moments, the immediate demise of capitalism, not unlike the vanquishing of financial uncertainty that caused the financial crisis, seemed very real indeed. Holding on to that belief exorcises the painful thoughts about the evictions, the job losses, the hunger, and the death. These losses and the powerful feelings they cause are the sources of such manic responses like the bombastic claims reported by Paul Mason (2009) and the “cautious joy” described by the Midnight Notes Collective (2009). In those moments, sections of the global justice movement constituted an all-powerful object onto which activists invested their hopes. Reality, and the repressed conflicts that prevented individuals and groups to relate to it, returned eventually and the global crisis became a crisis of the global justice movement too.

## Mourning Crises

Global crises raise important questions for the social sciences. What are they? What are the differences between, for instance, a financial crisis and a pandemic? Why does the denial of otherwise available knowledge prevent their prediction or the preventative development of sufficiently robust institutional defences? As seen above, basic assumptions in groups can escalate relatively quickly beyond control and cause self-destructive collective behaviours. These emotional dynamics build on the wish to avoid the anxiety caused by uncertainty and are exacerbated by individual and collective ambivalence towards object commitments. The incidence of these factors is further intensified by the speed of communication feedbacks in current times, by the shifting grounds of activists’ fluid networking dynamics, and by funders’ behaviour (and the related dynamics of dependence, envy and resentment). In these often unstable and sometimes volatile contexts, collective actions are subject to breakdowns that affect, and are affected by, the social and institutional systems within which they operate. At each step of the emotional sequences analysed above, appropriate institutional setups and interventions could arrest their development and prevent full-blown crises, or they could lessen their strength and mitigate their impact. The global justice movement has not yet developed robust enough institutions to embody past experience, to de-escalate spiralling social dynamics, and to address emotional accelerations. Such institutions could contribute to prevent the starkest effects of the kinds of path-dependent dynamics analysed in this article.

As we saw, in contexts of radical uncertainty, decision-making aims to reduce anxiety about the future. Decisions privilege good aspects of the objects on which they invest. These one-sided narratives spread within groups and in so doing they become increasingly self-evident, preventing challenges and alternative views. A sense of righteousness and euphoria prevails, which in turn causes an escalation of irrational beliefs. Eventually, external events make the lack of correspondence between narratives and group behaviour on the one hand, and reality on the other, of such magnitude that denial cannot be endured. Crisis reaches then its acute phase. Responses to group crisis follow two divergent paths. When faced with the consequences of biased decision-making and unrealistic belief systems, some actors respond to their initial dismay with blame. They reject personal responsibility and the need for reparation. Blame, however, prevents the working through of loss and, consequently, learning from experience and the pursuit of knowledge. Others turn blame on themselves as guilt. They acknowledge responsibility and attempt to repair the broken relationship with their (phantastic) objects. The prevalence of blame or guilt and the consequent attempt or refusal to repair the broken (individual or group) self, determine radically different crisis outcomes. The capacity to learn and the capacity to mourn are closely related (Bion, 1962; Klein, 1940). States of mind determine learning outcomes in individuals and groups. A prevalently BA mentality prevents mourning and learning. A prevalently W mentality allows attunement with reality, both internal to the group and external, and allows it to learn from its collective experience.

With no intention at either sweeping apology or blame, but observing different systemic dynamics – and mindful of how these diverging systems may be aspects of a wider whole displaying a critical structure – I highlight the following contrasting reactions to crisis. On the one hand, David Tuckett, interviewed in March 2020 following one of the most dramatic stock exchange crashes in history, reported how financial markets reacted to the chaos inflicted by Covid-19 with a business-as-usual attitude that excluded an analysis of the known systemic vulnerabilities of the global financial market to powerful shocks (Seibt, 2020). This attitude represents the uncompromising expression of externalisation of responsibility as blame (this time onto the Covid-19 virus). On the other hand, confront this with WSF activists’ responses to their own crisis. They internalised failure and turned it into self-berating attitudes that led, eventually, to proposals for dramatic institutional restructuring (even institutional euthanasia). Crises may be both inevitable and unforeseeable. Reactions can, however, vary and reflect alternative group cultures. The differences between Wall Street and global justice activists are stark.

Repeated boom-bust cycles, from exhilaration to shock and dismay, generate habituation to repeated crises and fatalistic attitudes to them. This may cause the entrenchment of beliefs about the impossibility to change what are perceived as fixed (or subject to slow immanent change) social structures or constitutional predispositions. This is what I refer to as critical structures. This may induce individuals and groups to defeated acceptance of that which is beyond the reach of individual and collective agency. Emancipation becomes an illusion of redemption projected into a utopian future or the afterlife. Even more destructively, it becomes a delusion, an escape from one’s present predicaments into fantasies of radical and quick change. The step from disillusionment to feelings of powerlessness and irrelevance, to individual and group abandonment, to the investment in authoritarian saviours is not long. Whether crises are inherent in dynamics of social change, their intensity and recurrence are not predetermined. Blaming, including human nature and social structures, fosters fatalism and prevents learning. Instead, collectively confronting and accepting the loss of what is no more (or never was) allows openness to what is not yet. This cannot prevent crises, but it may prevent compulsive repetitions and foster development.

Global justice activists contribute to deepen and extend our understandings of crisis beyond inevitable recurrences in immanent social processes. They do so by highlighting how crises have inherent political attributes and unequal effects depending on power differentials between social actors. For instance in a blame-laden, prevalently BA large group situation, the qualities of dependence and vulnerability inherent to the human condition are politically removed from shared global ownership and are forced onto the poor, refugees, black people, women. These groups are then subjected to disparagement, exploitation and political domination. That is the section of the world’s population for whom world activists mobilise. These are the people blamed for their laziness, inferior constitutional endowment and self-destructive behaviour, deemed to cause them to be ravaged by the consequences of financial crises and pandemics. These dynamics begin to describe the social embodiment of crisis into impervious critical structures. Global activists claim that the externalisation of crises onto the weak and vulnerable, as well as onto the environment, is globally self-destructive. We are all, indeed, in this together and exploitative behaviour vis-à-vis others and the environment causes devastating consequences for all.

As I write, the WSF is experiencing a resurgence of activity and commitments. As Covid-19 ravages the world, affecting the most vulnerable disproportionately, the current main drive is to promote its alternative vision for the post-pandemic world. Paramount among its activists is the intent to consolidate past connections between social movements across the planet and to develop new ones with the movements that are shaping the present political debates, including the environmental, antiracist, LGBTQ and feminist movements. A virtual WSF convened in January 2021 and its outcome gave rise to moderate optimism about the resilience of the project. However, a general sombreness could be perceived in the activists’ mood as a consequence of the combined effects of the pandemic, the isolation, the uncertainty, and the agentless nature of the threat. This sombreness seems congruent with a collective mourning not only of the lives lost and the livelihoods destroyed, but of the potential loss of a lifestyle that affects, to a greater or lesser extent, almost everyone on the planet. Given the circumstances, however, and the contingent difficulties to organise collective political actions and to influence the debate on the pandemic, this somberness might acquire melancholic aspects. The main challenge faced by global activists is to avoid both extremes of blame and melancholia (Freud, 1917/1957). The global justice movement has a tendency to turn loss of its objects (organisations and political projects) into melancholia (Nunes, 2018; Traverso, 2016; Brown, 1999; Benjamin, 1931/1974). Melancholia impacts the ability to work together and can ignite further fragmentation and mistrust, and self-destructive behaviour. Internal conflicts and divisions offer themselves as the target of blame if the hoped-for resurgence of the WSF fails to materialise. In case of failure, the internal other can be blamed and, eventually, the whole project can be disparaged as self-righteousness prevails.

Activists – in the WSF, in the larger global justice movement, and historically – often notice their difficulties in learning from experience, in institutionalising knowledge, and in embodying the principles they advocate. This generates dispiriting repetitions of failure. As illustrations of alternative approaches to this difficulty, consider two moments in the global justice movement. On the one hand, the funerals for the death of capitalism following the onset of the financial crisis of the late 2000s constituted split appraisals of the complexity of reality sustained by the belief that capitalism was actually dead. On the other hand, the pained invitation to let the IC die expressed an acceptance of the loss of a dear object. Whitaker's words warned of the futility of clinging to a form of global activism and the importance to mourn it instead in order to retain its spirit. If this loss can be mourned, the creative energy that was invested in the dead object would become available for new global political action.

## Endnotes

1. For psychoanalytically informed social movement research see Langman and Benski (2019) and Ormrod (2007).
2. All quotes are quasi-verbatim as recorded by me at the meetings. The speakers will remain anonymous. I identify them only by region and gender to provide some context. By de-emphasising their individual political perspective and multiple social identities, I do not wish to obscure their importance in social analysis. Rather, when powerful emotional dynamics of the kind I study here prevail, a culture of de-politicisation and de-differentiation, takes over. In those moments, differences are elided and diversity is used defensively. The 28 quotes are numbered and the speakers are as follows (the city indicates the location where the statement was made): 1, European man; 2, African woman; 3, Latin American woman; 4, 17 and 23, Latin American man; 5, 12 and 25 Asian man; 6, European woman; 7, European woman; 8, African woman; 9. European woman; 10 African man; 11, 19 and 20, European man; 13, man (my notes do not identify him); 14, 15 and 21, European man; 16, European man; 18, man (my notes do not identify him, not the same person as 13); 22, Latin American woman; 24, European woman; 26, Asian man; 27 and 28, Latin American man. Seventeen quotes are by men and eight by women, twelve by European, seven by Latin American, four by Asian and three by African activists. These figures roughly reflect activists’ participation to the IC meetings.
